# Governing through imaginaries: on the place and role of constructions of Japan within UK policy discourse regarding science, technology, and innovation

**DOI:** 10.1093/jlb/lsaa007

**Published:** 2021-08-16

**Authors:** Martyn Pickersgill

**Affiliations:** Centre for Biomedicine, Self and Society, The University of Edinburgh, Edinburgh EH8 9YL, UK

**Keywords:** sociotechnical imaginary, science, technology, innovation, research and development (R&D), Japan

## Abstract

This article employs the notion of ‘sociotechnical imaginaries’ from the discipline of science and technology studies in order to consider the role that constructions of Japan play within UK policy discourse on science, technology, and innovation. The analysis is parsed in relation to the three dominant constructions that emerged from within the discourse under study: Japan as collaborator, as comparator, and as competitor. The mentioning of Japan within policy texts seems often aimed at evoking an imaginary of an economically successful and technoscientifically inventive nation, geared up for investment and innovation. Japan was present in the texts analyzed as a country that was simultaneously the same and other to the UK: similar enough for meaningful comparisons to be made, while sufficiently different to motivate the UK to ‘do better’ and to galvanize symbolic and material resource to become ‘more like’ Japan. Thus, a sociotechnical imaginary emerged that was at once familiar to and yet also distinct from that of the UK. Sociotechnical imaginaries of other/Other nations can govern through enabling and shaping political and policy conversations, which can ultimately inflect and indeed help to determine different forms of legal and regulatory tools, processes, and discourses.

## I. INTRODUCTION

The social scientific concept of imaginaries, McNeil and colleagues argue, ‘seems to offer new ways to investigate the relationships among science, technology, and society’.^1^ Accordingly, its use has proliferated within literatures in science and technology studies (STS), anthropology, and—as this special issue testifies to—law.^2^ Here, I draw insight from STS scholars Sheila Jasanoff and Sang-Hyun Kim’s particular notion of ‘sociotechnical imaginaries’ in order to think through the role imaginaries of Japan play within UK policy discourse on science, technology, and innovation. These thoughts in turn serve as a provocation for concluding reflections on how imaginaries might govern.^3^

Science, technology, and innovation have been keenly emphasized within UK policy, and so discourse around these matters represents a useful site from which to begin to reflect upon the role of imaginaries in governance.^4^ In regard to Japan, as historian Gregory Clancey writes, the nation ‘sustains both technical and social momentum over a wide range of innovations and practices’.^5^ Japan is a friend to, but not a geographic or linguistic neighbor of, the UK, and this same/other dynamic plays out intriguingly within the policy and related discourse of the latter nation. The analysis of imaginaries of Japan that are produced within the UK thus provides a compelling starting point for what is hoped will be future considerations of how sociotechnical imaginaries contour and impact political and policy discourses and their legal and regulatory ramifications. More generally, this paper also aims to further develop emerging rapprochements between STS and law.^6^

Through the term ‘sociotechnical imaginary’, Jasanoff and Kim seek to describe ‘collectively imagined forms of social life and social order reflected in the design and fulfilment of nation-specific scientific and/or technological projects’.^7^ These entail visions not only of what is possible, but also—as Jasanoff writes—‘how life ought, or ought not, to be lived’.^8^ Researchers attendant to a variety of concerns have employed the concept, such as in studies of global health, biomedical research and development (R&D), and the application of neurotechnologies.^9^ Imaginaries, Jasanoff asserts, ‘can originate in the visions of single individuals or small collectives, gaining traction through blatant exercises of power or sustained acts of coalition building’.^10^ As Kim notes, ‘institutions of governance’ are particularly adept at propelling them.^11^ While most analyses of imaginaries have focused on how particular nations imagine themselves, the analysis advanced through this paper is concerned with how imaginaries of other nations are constructed as part of how intra-national discourse governs its own ends.

To do so, I draw on publicly available UK policy documents pertaining in some way to Japan that are located on the www.gov.uk portal. I specifically searched for documents produced between 2010 and 2019 within the topic area of ‘Business and industry’, and the sub-topic ‘Science and innovation’. This yielded 89 results, and all documents—which included reports, speeches, press releases, and news stories—were inspected for how Japan was discussed. This sample cannot, of course, be deemed to represent an exhaustive list of all relevant documents produced by the UK government or its quangos during this timeframe. It does, however, provide an indication of how Japan—particularly its practices and policies regarding science, technology, and innovation—has been constructed within UK policy discourse. Accordingly, it is a sufficient basis upon which reflections can be made around the role that sociotechnical imaginaries of Japan might play in governing how the UK is imagined and acted upon by its politicians and policy actors.

In what follows, I outline the data collected and advance considerations of this, parsing the analysis in relation to the three dominant (and partly overlapping) constructions of Japan that emerged from within the discourse under study. These are Japan as collaborator, as comparator, and as competitor. This article concludes with a summary of the analysis and with reflections on the role sociotechnical imaginaries of other (or, indeed, Other) nations might play within domestic governance.

## II. IMAGINING JAPAN

### II.A. Collaborator

In some discourse from the (now defunct) Department for Business, Innovation and Skills (BIS), and in particular the British Embassy Tokyo, Japan was winsomely presented as a current or future collaborator with the UK. One British Embassy Tokyo news story, for instance, emphasized how the UK and Japan ‘have a long history of learning from each other’, with another stating that when ‘the UK and Japan work together, we are world leaders’.^12^^,^^13^ In a 2011 press release pertaining to the UK-Japan Joint Committee on Co-Operation in Science and Technology, existing collaborations between the nations were noted, and ‘the possibilities of expanding collaboration into new areas dealing with the major challenges of global issues’ were highlighted.^14^ Sometimes collaborative agendas were reported as legalistically inscribed in treaties, such as the UK/Japan Agreement concerning Transfer of Arms and Military Technologies (TS No.27/2013) or within a Memorandum of Cooperation (MoC).^15^^,^^16^ Specifically, an MoC in 2017 between the UK Medical Research Council and the Japan Agency for Medical Research and Development was framed as promoting ‘research collaboration in areas of medical science that build on the strengths of both countries’, including in regenerative medicine.^17^ Collaboration was also encouraged through, for instance, a booklet detailing sources of funding that could support UK–Japan collaboration in science and innovation.^18^ Other policy documents underscored how, with UK governmental support, monies for UK research and development (R&D) were secured from Japanese investors.^19^

Reflecting the diplomatic functions of Embassy communications, news items from the British Embassy Tokyo commonly presented Japan as a country from which the UK might learn through collaborations. For example, one 2013 news story described a visit to Japan by Douglas Kell, Chief Executive Officer of the UK Biotechnology and Biological Sciences Research Council. The story suggested that collaboration between the UK and Japan would afford benefit to both nations.^20^ Specifically, it detailed how visits made within Japan granted the UK ‘delegation an insight into how Japan is addressing the challenges of scaling up research into application, and how industrial biotechnology in Japan integrates disciplines and brings together skills from a range of areas’.^21^ Similarly that year, technology was flagged in a missive from the Embassy as a ‘strength’ in Japan, in a narrative around UK–Japan engagements concerning neuroengineering.^22^ In 2014, the Embassy reported on a symposium to consider how Japan and Britain—both ‘world leaders’ in stem cell science—‘can work together to help deliver the huge promise of new cell therapies’.^23^ The possibilities of exciting collaborations in ‘health and life sciences’ were again described in 2016.^24^ Much, then, has been made of the (potential for) collaboration between Japan and the UK in relation to science and technology, including specifically biomedicine and health R&D.

In order for a collaboration to be feasible, it requires some kinds of similarities between actors, institutions, and other entities involved in and enabling it. Characterizing collaborators or calling for collaboration can also be performative practices. By this, I mean that to call on X and Y to collaborate is a means of not merely describing ontological resonance, but a technique to actively craft these ontologies—at least at the level of rhetoric.^25^ In the case at hand, discussions of UK–Japan collaborations in UK policy discourse construct scientific research and the policies supporting it within Japan as being sufficiently resonant for collaboration to be feasible. In so doing, a sociotechnical imaginary of Japan as having key similarities with—as well as strengths compared to—the UK is performed. In what follows, I focus on discourses of comparison in particular.

### II.B. Comparator

In the sample of documents inspected for this analysis, policy discourses of collaboration sometimes involved latent comparison; other texts, though, more explicitly cast Japan and the UK as comparators. Through presenting comparisons, these documents also positioned the nations as similar in relation to science and industry, but with some salient differences that played various rhetorical roles. On occasion, comparisons were aimed at portraying the UK in a positive light; for instance, a 2010 news item produced by BIS noted that ‘78% of global R&D occurs in five countries: the US; Japan; Germany; France and the UK’.^26^ R&D is commonly linked to notions of technoscientific and societal progress, and so the UK’s position in this ‘top five’ can be seen to be affirming a construction of this nation—and the others in the list—as progressive and forward-looking.^27^ A few months later, an oral statement to Parliament by Vince Cable MP of BIS again noted the commonalities between the UK and ‘other industrialised countries’, once more citing the USA, France, and Germany.^28^ The sense that Japan and the UK are part of special, small group of similar nations is clearly conveyed through these comparisons, in ways that are flattering to the UK.

Japan was also related to the UK in more specific cases, such as low carbon vehicle R&D, where the UK and Japan (alongside the USA and Germany) were positioned as ‘world leaders’.^29^ In the case of big data analytics (for instance, to improve healthcare), a press release from BIS et al. on a £73 million investment in this area highlighted in its ‘Notes to editors’ that Japan has already allocated ‘nearly £90 million for big data R&D’. ^30^ This suggested similarities between the industrial policies and R&D practices between the countries, as well as a legitimation of the UK largess by implying a need to ‘catch up’ with Japan. In a speech by then Chancellor of the Exchequer George Osborne MP, examples were given of research conducted by the UK that was then ‘exploited’ into technology manufacture by ‘countries like Japan and Switzerland’.^31^ This example fed into a narrative about the need for a ‘long term plan for science’ as ‘part of our economic plan’.^32^ The UK, then, was accounted for as being better served by being more like Japan. This theme was also apparent in a 2014 speech given by Secretary of State for Business, Innovation and Skills Vince Cable MP, where UK investment in R&D was presented as needing—in ‘a globally competitive environment’—to be closer in percentage GDP to ‘our comparators’, among which was Japan.^33^ Implicit in Cable’s and especially Osbourne’s speeches was the notion that care needed to be taken such that the UK would not ‘lag behind’ Japan. Accordingly, sociotechnical imaginaries of the UK and Japan were set up that were partly isomorphic, but also mimetic—with the former judged to require evolution into the latter.

Sometimes, a desire to imitate and a sense of pride in ‘outdoing’ Japan were simultaneously evident across different yet interrelated texts. In particular, a 2011 news story from BIS and Minister for Universities and Science David Willetts MP underscored that the UK ‘has more articles per researcher, more citations per researcher, and more usage per article authored than researchers in US, China, Japan and Germany’.^34^ At the same time, an oral statement to Parliament from BIS and Willetts made on the same day (October 19, 2011) also flagged that ‘more papers are co-authored with industry in Japan, the US, Germany and France than in the UK’.^35^ Apparently, ‘we, in Britain, are great inventors, responsible for so many scientific and intellectual breakthroughs and yet [we] don’t always exploit them as much as we might’. In contrast, then, Japan was cast as a country where engagements between scientific research and private industry were more common and which should be emulated in the UK.^36^

These documents thus partly blurred ‘comparator’ with ‘competitor’. Other texts were more explicit in this regard: in 2015, for instance, a speech by Business Secretary Sajid Javid MP on ‘the government’s commitment to innovation’ proudly noted ‘that Britain has now reached second spot in the Global Innovation Index, ahead of the USA, Japan, and every other nation in the EU’.^37^ In 2017, Science Minister Jo Johnson MP made another comparison between the UK and ‘comparable countries’, which painted the former in a positive light: specifically, in the UK, ‘a far greater proportion of R&D – 26% – takes place in our universities’, as compared ‘with 20% in France, 17% in Germany, 13% in the US and 12% in Japan’.^38^

Similar to calls for collaboration, characterizations of different nations as comparators are also forms of ontological work: the act of comparing can have the effect of helping to make things (seem) comparable.^39^ In this regard, sociotechnical imaginaries of Japan and the UK—as industrialized ‘world leaders’ in science and technology, where innovation is ostensibly supported and fostered—are enacted and made resonant. It is precisely because of these resonances that both clear and latent discourses of competition can emerge, the analysis of which this paper now turns.

### II.C. Competitor

As some of the writings discussed above begin to indicate, Japan has been presented within policy discourse as being in ‘competition’ with the UK. On rare occasions, this was explicit within the texts under consideration: for instance, in a 2011 Oral statement to Parliament, David Willetts MP described how ‘the UK produces more science graduates per 100,000 employed people in the 25- to 34-year-old age bracket than many of our competitors’, with Japan listed as one of these.^40^ Generally, though, the UK was configured as being in what I call ‘cryptic competition’ with Japan. By this, I mean that competition was thematically present, yet not directly referred to: the word ‘competitor’ itself did not appear in many texts, but rather was made visible through the tenor and tone of writings and the context within which words like ‘comparator’ were used. The use of ‘comparator’ in such discursive milieu belied its formal meaning and instead conveyed to the reader that it was being used in the place of the potentially less seemly ‘competitor’—while connoting almost the same.

The 2011 International Comparative Performance of the UK Research Base was a key report within which Japan was implicitly—never explicitly—regarded as a competitor.^41^ The report was produced for BIS by publisher and analytics company Elsevier. In it, Japan and various other nations were termed ‘comparator’ nations. Yet, since the entire report was aimed at showcasing and discussing rankings and metrics in which it appears to be desirable to ‘do well’, it is challenging to read the word ‘comparator’ within the report as anything but a euphemism for ‘competitor’. Within the report, comparators/competitors like Japan were not always presented in the most affirming fashion, with the extract below being the most vivid example of an unflattering portrayal of Japanese culture, science, and citizens:

‘It is of note that Japan, one of the largest research nations in the world, does not feature more prominently in the lists of source of destination countries for UK researchers [sic]. This may support earlier views that Japan runs an “intellectual closed shop” [in-text citation], characterised by a large proportion of ‘stay-at-home’ researchers and high return rates from abroad [in-text citation]’.^42^

This extract resonates with UK stereotypes of Japanese citizens as closed and secretive.^43^ It offers an explanation of relatively low migrations of UK researchers to Japan as an epiphenomenon of the culture of that nation, rather than that of the UK. It also completely elides (among other things) the roles of law and regulation in shaping the possibilities and practicalities of migration. Accordingly, these two sentences contributed within the report to explicitly casting a particular sociotechnical imaginary of Japan as being overly concerned with its own domestic and scientific affairs and as existing in isolation from the ostensibly outward-looking policies and practices of innovation of other nations (in implied contrast to the UK). An almost identical set of sentences appeared within the 2013 edition of the report (with ‘stay-at-home’ becoming ‘Sedentary’): ‘Japan’s very high proportion of Sedentary researchers appears to confirm the view that Japan runs an “intellectual closed shop”’.^44^ In the 2016 iteration, similar words were used again, with ‘Sedentary’ morphing into the less pejorative ‘Non-migratory’.^45^ Whatever, exactly, an ‘intellectual closed shop’ might be, it seems that this is a characterization that is resistant to removal.

Some other language in the 2011 report further suggested a scientific culture within Japan that self-consciously ignores the wider world and where the UK was indirectly portrayed as a significant actor. Specifically, the report authors detailed the results of a citation analysis of scientific articles and concluded that Japan (like the USA and China) is ‘strongly under-citing UK articles’.^46^ Later in the report, Japan was asserted to have (like China and Germany) ‘a very different research strength profile’ to the UK. Japan and Germany were, it seems, ‘very focused in physics and chemistry’, but ‘neither have significant strengths in the social sciences’.^47^ In contrast, ‘the UK has strengths in all of the major areas of research’.^48^ Similar claims were advanced in the 2013 iteration of this document (p. 50).^49^ The 2011 report flagged Japan (with the USA, China, and Germany) as having patent application rates that were higher than the UK, but this was suggested as being ‘at least in part’ the result of ‘differences in field specialisation’. Differences in patent law and regulation between Japan and the UK were not discussed, nor were how different forms of governance and flows of professional and financial capital might incentivize patent applications in differing ways.

The fact that the UK filed ‘fewer patents’ than Japan was later noted in a 2013 Foresight report on The Future of Manufacturing, in a 2014 speech by Chancellor George Osborne MP, and in a 2016 speech by Secretary of State for Business, Energy and Industrial Strategy Greg Clark MP.^50^^,^^51^^,^^52^ In the Foresight report, Japan was explicitly flagged as a ‘competitor’ nation. For example, governmental investments in e-infrastructure were highlighted as necessary ‘to ensure UK businesses are not left behind those of competitor nations’ like Japan as well as the USA and China.^53^ Decreased manufacturing output growth in the UK compared to Japan was reported (p. 114), as well as the fact that a smaller percentage of UK manufacturing firm managers had university degrees as compared to Japan (p. 174). This latter point related to a claim that the UK ‘currently fares poorly, when compared internationally, on the quality of its managers’.^54^ Different varieties of capitalism between the UK and Japan were invoked to explain some of the differences noted between the nations:

‘For investment generally, and R&D in particular, it has been argued that the UK variety of liberal market capitalism inhibits long-term investment compared with more coordinated varieties exemplified by Germany and Japan [in-text citation]’.^55^

Alongside these more analytic reflections were somewhat speculative ruminations about the ‘national psyche’ of ‘more successful economies’ than the UK.^56^ This included Japan, where ‘it appears that a value creation ‘mind-set’ is embedded in the national psyche’.^57^ The report consequently constructed not merely a sociotechnical imaginary of Japan, but what we might term a psychotechnical imaginary as well.

In contrast to the aforementioned 2011 BIS document, the 2013 Foresight Report made several links to law and regulation—particularly in relation to financial law and the need for environmental regulations to enhance sustainability. In the section of the document which detailed its implications for the UK Government, the first of these was as follows:

‘Greater use should be made of well designed regulation, in particular drawing upon ideas from abroad. For example, effective energy reduction has been demonstrated by innovative schemes such as ‘Top-Runner’ in Japan [in-text citation] where future product standards are set so that all products manufactured at a specific point in the future must be at least as good as the best performance of today. The Government should consider developing top-runner schemes in the UK’.^58^

The UK, then, was indicated as having the potential to afford benefit from attending to and potentially mimicking legislative experiments within its ‘competitor’ Japan.

As compared to the total sample of policy texts inspected for this article, though, these specific and more considered discussions of law and regulation were a relative anomaly. The aforementioned elisions regarding law in discussions of patents within the 2011 Elsevier report for BIS (and subsequently) were congruent with the contents of much of that document, wherein law and regulation made barely any appearance. This absence occurred to quite some extent in the wider discourse of BIS concerning Japan, which very rarely explicitly acknowledged the role and impact of legal and regulatory tools in shaping the sociotechnical contexts within which R&D occur. This is despite the evident import of these for promoting particular kinds of innovation.^59^

In sum, UK policy texts often presented Japan implicitly or explicitly as a ‘competitor’ nation: as an entity that should be, could be, or had been successfully challenged or which could be learned from in order to achieve this. At the time, the role of law and regulation in patterning and propelling difference remained a minor feature of the discourse. By generally underplaying legal and regulatory approaches, techniques, and conventions, exactly how these diverged between the Japan and the UK was largely downplayed—suggesting little or insignificant differences (with the notable exception of the 2014 report, which makes the absences elsewhere all the more striking). Consequently, the sociotechnical imaginary of Japan built through the texts under study was ambiguously, indeed ambivalently, different to yet somehow almost the same as that of the UK.

## III. CONCLUSION

Within the texts analyzed, Japan was often mentioned only fleetingly; for example, as a comparison point around investment. But fleeting mentions nevertheless can carry considerable rhetorical weight, and the carefully crafted concision of political speeches means that references to other nations can hardly be considered accidental or unimportant. As scholarship in traditions such as critical race theory indicates, mentions of the word ‘Japan’ also carry with them an *idea* of Japan.^60^ This is contoured through the wider context and content of the text, as well as through internationally circulating notions of the country that convey and animate effects of intrigue and assumptions of technological sophistication.^61^ The mentioning of Japan within policy texts thus seemed often aimed at evoking an imaginary of an economically successful and technoscientifically inventive nation, geared up for investment and innovation.

Japan was present in the texts analyzed in this essay as a country that was simultaneously the same and other to the UK: similar enough for meaningful comparisons to be made and sufficiently different to motivate the UK to ‘do better’ and to galvanize symbolic and material resource to become ‘more like’ Japan. Thus, a sociotechnical imaginary emerged that was at once familiar to and yet also distinct from that of the UK. In practice, regulatory moves made in Japan in relation to supporting regenerative medicine, for instance, have helped to enjoin similar transitions in various countries within ‘the West’.^62^

Nested within the sociotechnical imaginary presented in some documents was a psychotechnical imaginary. There, imaginaries of citizen dispositions and attitudes were dynamically linked to particular economic and technoscientific trajectories, such as in talk of a ‘national psyche’ and how that inflected R&D. This imaginary leveraged longstanding stereotypes of the ‘inward’ looking nature of Japan to celebrate the purported openness of the UK.

It is in this sense, among others, that imaginaries govern. The sociotechnical imaginaries of collaborator/comparator/competitor nations assembled within policy discourse can reinforce particular constructions and trajectories of the organizations and individuals enunciating these. In many cultural contexts it is, in part, through constructions of the Other that constructions of the self are sustained.^63^ Imaginaries can also act to propel forward new, or newly intensified, agendas—such as technocratic imperatives to innovate, innovate, innovate. When a senior politician, for instance, describes features of another nation in admiring, even envious, tones, or makes comparisons in ways that affirm aspects of their own country, these assertions and reflections are not innocent. Rather, they signal what powerful actors consider the state in question *should* be like; sometimes so too do assertions of what it *is* like, where purported description serves as an implicit normative proclamation. Sociotechnical imaginaries of other/Other nations thus govern, I suggest, through enabling and shaping political and policy conversations, which can ultimately inflect and indeed help to determine different forms of legal and regulatory tools, processes, and discourses.

### FUNDING STATEMENT

This research was funded by the ESRC-AHRC [ES/S013873/1] and the Wellcome Trust [106612/Z/14/Z; 209519/Z/17/Z].

## Footnotes

1 Maureen C. McNeil et al., *Conceptualizing Imaginaries of Science, Technology and Society*, in The Handbook of Science and Technology Studies 435 (Ulrike Felt et al. eds., 2016).

2 Mark Flear and Richard Ashcroft, *Special Issue Introduction*; Mark L. Flear and Thomas Pfister, *Contingent Participation: Imaginaries of Sustainable Technoscientific Innovation in the European Union*, in Knowledge, Technology and Law (Emilie Cloatre and Martyn Pickersgill eds., 2014); Martyn Pickersgill, *Connecting Neuroscience and Law: Anticipatory Discourse and the Role of Sociotechnical Imaginaries*, 30 New Genetics and Society 27 (2011).

3 Sheila Jasanoff and Sang-Hyun Kim, *Containing the Atom: Sociotechnical Imaginaries and Nuclear Power in the United States and South Korea*, 47 Minerva 119 (2009).

4 John Agar, Science Policy Under Thatcher (2019).

5 Gregory Clancey, *The History of Technology in Japan and East Asia*, 3 East Asian Science, Technology and Society 525 (2009), at 528.

6 Emilie Cloatre, Pills for The Poorest: An Exploration of Trips and Access to Medication in Sub-Saharan Africa (2013); Emilie Cloatre and Martyn Pickersgill Eds., Knowledge, Technology and Law (2014); Christopher Lawless and Alex Faulkner Eds., Material Worlds: Intersections of Law, Science, Technology, and Society (2012); Kate Seear, Law, Drugs, and The Making of Addiction (2020).

7 Jasanoff and Kim, *supra* note 3, at 120.

8 Sheila Jasanoff, *Future Imperfect: Science, Technology, and the Imaginations of Modernity*, in Dreamscapes of Modernity: Sociotechnical Imaginaries and The Fabrication of Power (Sheila Jasanoff and Sang-Hyun Kim eds., 2015), at 4.

9 Sang-Hyun Kim, *The Politics of Human Embryonic Stem Cell Research in South Korea: Contesting National Sociotechnical Imaginaries*, 23 Sci. as Cul. 293 (2014); Andrew Lakoff, *Global Health Security and the Pathogenic Imaginary*, in Dreamscapes of Modernity: Sociotechnical Imaginaries and the Fabrication of Power (Sheila Jasanoff and Sang-Hyun Kim eds., 2015); Martyn Pickersgill, *Connecting Neuroscience and Law: Anticipatory Discourse and the Role of Sociotechnical Imaginaries*, 30 New Gen. Soc. 27 (2011); Elta Smith, *Corporate Imaginaries of Biotechnology and Global Governance: Syngenta, Golden Rice, and Corporate Social Responsibility*, in Dreamscapes of Modernity: Sociotechnical Imaginaries and the Fabrication of Power (Sheila Jasanoff and Sang-Hyun Kim eds., 2015); Neil Stephens, Paul Atkinson and Peter Glaser, *Institutional Imaginaries of Publics in Stem Cell Banking: The Cases of the UK and Spain*, 22 Sci. as Cul. 497 (2013).

10 Jasanoff, *supra* note 8, at 4.

11 Sang-Hyun Kim, *The Politics of Human Embryonic Stem Cell Research in South Korea: Contesting National Sociotechnical Imaginaries*, 23 Sci. as Cul. 293 (2014), at 296.

12 British Embassy Tokyo, *Creating the Future—Journeys in Engineering from UK to Japan*,
https://www.gov.uk/government/news/creating-the-future-journeys-in-engineering-from-uk-to-japan (accessed Nov. 19, 2019).

13 British Embassy Tokyo, *What is the Secret to Innovation Success? Experience from the UK*, https://www.gov.uk/government/news/what-is-the-secret-to-innovation-success-experience-from-the-uk (accessed Nov. 19, 2019).

14 Department for Business, Innovation and Skills and Government Office for Science, *Eighth Meeting of the UK-Japan Joint Committee on Co-Operation in Science and Technology*, https://www.gov.uk/government/news/eighth-meeting-of-the-uk-japan-joint-committee-on-co-operation-in-science-and-technology (accessed Nov. 19, 2019).

15 Foreign and Commonwealth Office, *UK/Japan: Agreement Concerning Transfer of Arms and Military Technologies [TS No.27/2013]*, https://www.gov.uk/government/publications/ukjapan-agreement-concerning-transfer-of-arms-and-military-technology (accessed Nov. 19, 2019).

16 British Embassy Tokyo and UK Science and Innovation Network in Japan, *New UK-Japan Collaboration on Medical Research and Development*, https://www.gov.uk/government/news/new-uk-japan-collaboration-on-medical-research-and-development accessed (accessed Nov. 19, 2019).

17 British Embassy Tokyo and UK Science and Innovation Network in Japan, *New UK-Japan Collaboration on Medical Research and Development*, https://www.gov.uk/government/news/new-uk-japan-collaboration-on-medical-research-and-development (accessed Nov. 19, 2019).

18 Foreign and Commonwealth Office, *UK-Japan Science and Innovation Collaboration: Sources of Funding, 2014*, https://www.gov.uk/government/publications/uk-japan-science-innovation-collaboration-sources-of-funding-2014 (accessed Nov. 19, 2019).

19 Foreign and Commonwealth Office and Department for Business, Energy and Industrial Strategy, *SIN Japan Leads GREAT Robotics Campaign Resulting in Over £350,000 of Investment*, https://www.gov.uk/government/publications/sin-japan-leads-great-robotics-campaign-resulting-in-over-350000-of-investment (accessed Nov. 19, 2019).

20 British Embassy Tokyo, *Designing a Future Fit for an Ageing Society*, https://www.gov.uk/government/news/design-for-an-aging-society-transport-systems-and-technologies (accessed Nov. 19, 2019).

21 British Embassy Tokyo, *Bioscience Collaboration: Research for Food, Pharma and Fuel*, https://www.gov.uk/government/news/bioscience-collaboration-research-for-food-pharma-fuel (accessed Nov. 19, 2019).

22 British Embassy Tokyo, *UK and Japanese Scientists Share Secrets of the Brain*, https://www.gov.uk/government/news/uk-and-japanese-scientists-share-secrets-of-the-brain (accessed Nov. 19, 2019).

23 British Embassy Tokyo, *Japan and Britain: Stem-Cell Pioneers*, https://www.gov.uk/government/news/japan-and-britain-stem-cell-pioneers (accessed Nov. 19, 2019).

24 British Embassy Tokyo and UK Science and Innovation Network in Japan, *10th Meeting of the Japan-UK Joint Committee on Co-Operation in Science and Technology*, https://www.gov.uk/government/news/10th-meeting-of-the-japan-uk-joint-committee-on-cooperation-in-science-and-technology (accessed Nov. 19, 2019).

25 John Law, *After ANT: Complexity, Naming and Topology*, 47(S1) Soc. Rev. 1 (1999); Annemarie Mol, *Ontological Politics. A Word and Some Questions*, 47(S1) Soc. Rev. 74 (1999).

26 Department for Business, Innovation and Skills, *R&D Spend of Leading UK Companies Holds Up During Downturn*, https://www.gov.uk/government/news/r-d-spend-of-leading-uk-companies-holds-up-during-downturn (accessed Nov. 19, 2019).

27 John Agar, Science in the 20th Century and Beyond (2013).

28 Department for Business, Innovation and Skills and The Rt Hon Dr Vince Cable, *Manufacturing and Young People: “Perceptions Must Change”*, https://www.gov.uk/government/speeches/manufacturing-and-young-people-perceptions-must-change (accessed Nov. 19, 2019).

29 Department for Business, Innovation and Skills, *Multi-Million Boost for UK Electric Vehicle Battery Technology*, https://www.gov.uk/government/news/multi-million-boost-for-uk-electric-vehicle-battery-technology (accessed Nov. 19, 2019).

30 Department for Business, Innovation and Skills, Arts and Humanities Research Council, Economic and Social Research Council, Medical Research Council, Natural Environment Research Council, and The Rt Hon David Willetts, *£73 Million to Improve Access to Data and Drive Innovation*, https://www.gov.uk/government/news/73-million-to-improve-access-to-data-and-drive-innovation (accessed Nov. 19, 2019).

31 Department for Business, Innovation and Skills, HM Treasury, and The Rt Hon George Osborne, *Chancellor of the Exchequer’s Speech on Science in Cambridge*, https://www.gov.uk/government/speeches/chancellor-of-the-exchequers-speech-on-science-in-cambridge (accessed Nov. 19, 2019).

32
*Id.*

33
*Id.*

34 Department for Business, Innovation and Skills and The Rt Hon David Willetts, *UK Research Base Most Productive in the World*, https://www.gov.uk/government/news/uk-research-base-most-productive-in-the-world (accessed Nov. 19, 2019).

35 Department for Business, Innovation and Skills and The Rt Hon David Willetts, *Gareth Roberts Science Policy Lecture*, https://www.gov.uk/government/speeches/gareth-roberts-science-policy-lecture--2 (accessed Nov. 19, 2019).

36
*Id.*

37 Department for Business, Innovation and Skills and The Rt Hon Sajid Javid, *Opening Up the Roads: The Government’s Commitment to Innovation*, https://www.gov.uk/government/speeches/opening-up-the-roads-the-governments-commitment-to-innovation (accessed Nov. 19, 2019).

38 Department for Business, Energy and Industrial Strategy and The Rt Hon Jo Johnson, *How Universities Can Drive Prosperity Through Deeper Engagement*, https://www.gov.uk/government/speeches/how-universities-can-drive-prosperity-through-deeper-engagement (accessed Nov. 19, 2019).

39 Richard Freeman and Steven Sturdy, *Doing Comparison: Producing Authority in an International Organization*, in The Politics of Expertise in International Organizations: How International Bureaucracies Produce and Mobilize Knowledge (Annabelle Littoz-Monnet, ed. 2017).

40 Department for Business, Innovation and Skills and The Rt Hon David Willetts, *Guardian HE Summit*, https://www.gov.uk/government/speeches/guardian-he-summit (accessed Nov. 19, 2019).

41 Elsevier, *International Comparative Performance of the UK Research Base—2011: A Report Prepared for the Department of Business, Innovation and Skills*, https://assets.publishing.service.gov.uk/government/uploads/system/uploads/attachment_data/file/32489/11-p123-international-comparative-performance-uk-research-base-2011.pdf (accessed Nov. 19, 2019).

42 Elsevier, *International Comparative Performance of the UK Research Base—2011: A Report Prepared for the Department of Business, Innovation and Skills*, https://assets.publishing.service.gov.uk/government/uploads/system/uploads/attachment_data/file/32489/11-p123-international-comparative-performance-uk-research-base-2011.pdf, at 23 (accessed Nov. 19, 2019).

43 Satoko Watanabe and Ryozo Yamaguchi, *Intercultural Perceptions at the Workplace: The Case of the British Subsidiaries of Japanese Firms*, 48 Hum. Rel. 581 (1995).

44 Elsevier, *International Comparative Performance of the UK Research Base—2013: A Report Prepared by Elsevier for the UK’s Department of Business, Innovation and Skills (BIS)*, https://assets.publishing.service.gov.uk/government/uploads/system/uploads/attachment_data/file/263729/bis-13-1297-international-comparative-performance-of-the-UK-research-base-2013.pdf, at 29 (accessed Nov. 19, 2019).

45 Elsevier, *International Comparative Performance of the UK Research Base 2016: A Report Prepared by Elsevier for the UK’s Department of Business, Energy and Industrial Strategy (BEIS)*, https://assets.publishing.service.gov.uk/government/uploads/system/uploads/attachment_data/file/660855/uk-research-base-international-comparison-2016.pdf (accessed Nov. 19, 2019).

46 Elsevier, *International Comparative Performance of the UK Research Base—2011: A Report Prepared for the Department of Business, Innovation and Skills*, https://assets.publishing.service.gov.uk/government/uploads/system/uploads/attachment_data/file/32489/11-p123-international-comparative-performance-uk-research-base-2011.pdf, at 42 (accessed Nov. 19, 2019).

47
*Id.*

48
*Id.*

49 Elsevier, *International Comparative Performance of the UK Research Base—2013: A Report Prepared by Elsevier for the UK’s Department of Business, Innovation and Skills (BIS)*, https://assets.publishing.service.gov.uk/government/uploads/system/uploads/attachment_data/file/263729/bis-13-1297-international-comparative-performance-of-the-UK-research-base-2013.pdf (accessed Nov. 19, 2019).

50 Foresight (The Government Office for Science), *The Future of Manufacturing: A New Era of Opportunity and Challenge for the UK*, https://assets.publishing.service.gov.uk/government/uploads/system/uploads/attachment_data/file/255922/13-809-future-manufacturing-project-report.pdf, at 21 (accessed Nov. 19, 2019).

51 Department for Business, Innovation and Skills, HM Treasury, and The Rt Hon George Osborne, *Chancellor of the Exchequer’s Speech on Science in Cambridge*, https://www.gov.uk/government/speeches/chancellor-of-the-exchequers-speech-on-science-in-cambridge (accessed Nov. 19, 2019).

52 Innovate UK, Department for Business, Energy and Industrial Strategy, and The Rt Hon Greg Clark, *A Place for Innovation*, https://www.gov.uk/government/speeches/a-place-for-innovation (accessed Nov. 19, 2019).

53 Foresight (The Government Office for Science), *The Future of Manufacturing: A New Era of Opportunity and Challenge for the UK*, https://assets.publishing.service.gov.uk/government/uploads/system/uploads/attachment_data/file/255922/13-809-future-manufacturing-project-report.pdf, at 141 (accessed Nov. 19, 2019).

54 Id. at 174; noted again on 182 (accessed Nov. 19, 2019).

55 Id. at 209 (accessed Nov. 19, 2019).

56 Id. at 215 (accessed Nov. 19, 2019).

57 Id. at 215 (accessed Nov. 19, 2019).

58 Id. at 28; noted again 163 (accessed Nov. 19, 2019).

59 Nayha Sethi, *Regulating for Uncertainty: Bridging Blurred Boundaries in Medical Innovation, Research and Treatment*, 11 Law, Inn. And Tech. 112 (2019).

60 Kimberlé Crenshaw, Neil Gotanda, Gary Peller and Kendall Thomas Eds., Critical Race Theory: the Key Writings That Formed the Movement (1995).

61 Koichi Iwabuchi, *Marketing ‘Japan’: Japanese Cultural Presence Under a Global Gaze*, 18 Japanese Stud. 165 (1998).

62 John Gardner, Andrew Webster and James Mittra, *The “Entrepreneurial State” and the Leveraging of Life in the Field of Regenerative Medicine*, in 25 Bioeconomies: Life, Technology, and Capital in the 21st Century (Vincenzo Pavone and Joanna Goven, eds., 2017).

63 Jong S. Jun, *The Self in the Social Construction of Organizational Reality: Eastern and Western Views*, 27 Ad. The. And Prax. 86 (2005).

